# Raloxifene reduces urokinase-type plasminogen activator-dependent proliferation of synoviocytes from patients with rheumatoid arthritis

**DOI:** 10.1186/ar1815

**Published:** 2005-09-08

**Authors:** S Guiducci, A Del Rosso, M Cinelli, F Perfetto, R Livi, A Rossi, A Gabrielli, R Giacomelli, N Iori, G Fibbi, M Del Rosso, M Matucci Cerinic

**Affiliations:** 1 Department of Internal Medicine, Division of Rheumatology, University of Florence, Florence, Italy; 2 Department of Experimental Pathology and Oncology, University of Florence, Florence, Italy; 3 Institute of Clinical Medicine, Hematology and Clinical Immunology, University of Ancona, Didactic pole, Torrette di Ancona, Ancona, Italy; 4 Department of Internal Medicine and Public Health, University of L'Aquila, L'Aquila, Italy; 5 Medical Direction, Eli Lilly Italia S.p.a., Sesto Fiorentino (FI), Italy

## Abstract

Extracellular fibrinolysis, controlled by the membrane-bound fibrinolytic system, is involved in cartilage damage and rheumatoid arthritis (RA) synovitis. Estrogen status and metabolism seem to be impaired in RA, and synoviocytes show receptors for estrogens. Our aims in this study were to evaluate in healthy and RA synoviocytes the effects of Raloxifene (RAL), a selective estrogen receptor modulator (SERM), on: proliferation; the components of the fibrinolytic system; and chemoinvasion. The effects of RAL were studied *in vitro *on synoviocytes from four RA patients and four controls. Proliferation was evaluated as cell number increase, and synoviocytes were treated with 0.5 μM and 1 μM RAL with and without urokinase-plasminogen activator (u-PA) and anti-u-PA/anti-u-PA receptor (u-PAR) antibodies. Fibrinolytic system components (u-PA, u-PAR and plasminogen activator inhibitor (PAI)-1) were assayed by ELISA with cells treated with 0.5 μM and 1 μM RAL for 48 h. u-PA activity was evaluated by zymography and a direct fibrinolytic assay. U-PAR/cell and its saturation were studied by radioiodination of u-PA and a u-PA binding assay. Chemoinvasion was measured using the Boyden chamber invasion assay. u-PA induced proliferation of RA synoviocytes was blocked by RAL (p < 0.05) and antagonized by antibodies alone. The inhibitory effect of RAL was not additive with u-PA/u-PAR antagonism. RA synoviocytes treated with RAL showed, compared to basal, higher levels of PAI-1 (10.75 ± 0.26 versus 5.5 ± 0.1 μg/10^6 ^cells, respectively; p < 0.01), lower levels of u-PA (1.04 ± 0.05 versus 3.1 ± 0.4 ng/10^6 ^cells, respectively; p < 0.001), and lower levels of u-PAR (11.28 ± 0.22 versus 23.6 ± 0.1 ng/10^6 ^cells, respectively; p < 0.001). RAL also significantly inhibited u-PA-induced migration. Similar effects were also shown, at least partially, in controls. RAL exerts anti-proliferative and anti-invasive effects on synoviocytes, mainly modulating u-PAR and, to a lesser extent, u-PA and PAI-1 levels, and inhibiting cell migration and proliferation.

## Introduction

It is well known that sex hormones are implicated in the immune response. Estrogens enhance humoral immunity, while androgens and progesterone are natural immune-suppressors [1]. In rheumatoid arthritis (RA), sex hormones fuel synovitis. Synovial macrophages, monocytes and lymphocytes [2] possess functional androgen and estrogen receptors and metabolize gonadal hormones [3]. In RA, an association of estrogen gene polymorphism with age at onset has been observed [4].

In both male and female RA patients, low levels of androgens and a low androgen/estrogen ratio have been reported [5]. This supports a possible pathogenic immunosuppressive role for decreased androgen levels. In RA, normal serum androgen and low estrogen levels, but high synovial fluid estrogen and lower androgen levels, indicate that peripheral sex hormone metabolism may be involved in the manifestations of the disease and seems to play an important role in the immune-inflammatory local response [6].

A recent study provides a link between estrogen receptors (ER-alpha) on fibroblast-like synoviocytes and regulation of extracellular matrix (ECM) functionality by the system of matrix metalloproteinases (MMPs)/tissue inhibitors of matrix metalloproteinases (TIMPs) [7]. The expression and activity of MMPs, as well as the levels of TIMPs, is stimulated by 17beta-estradiol, but inhibited by progesterone.

Excessive extracellular proteolysis characterizes neoplastic cell invasion [8], tumor- or inflammation-associated angiogenesis [9] and breakdown of the articular cartilage in osteoarthritis [10]. As well as MMPs, the cell-associated serine proteases of the plasminogen activator/plasminutes system are also involved in extracellular proteolysis required for cell invasion, possibly including cartilage and subcondral bone degradation in RA.

In the fibrinolytic system, the urokinase-type plasminogen activator (u-PA) interacts with its membrane receptor (u-PAR) and activates the single-chain proenzyme plasminogen to the two-chain broad-spectrum serine proteinase plasmin, which is able to degrade ECM both directly and indirectly through activation of secreted pro-MMPs.

Membrane-type MMP undergoes a plasmin-dependent activation that enables it to activate membrane receptor-bound progelatinase A, thus triggering a multienzyme cascade leading to ECM destruction and subsequent cell invasion [11]. Additionally, cell-associated proteases are required for the activity of pro-angiogenic factors, which sustain the synovial pannus growth [12].

Synovial cells express membrane u-PAR, and cultured RA synoviocytes display a higher production of plasminogen activator inhibitor (PAI)-1 than in osteoarthritis and normal synoviocytes [13], suggesting that the plasminogen activator/plasminutes system is involved in the inflammatory remodeling of connective tissues occurring in arthritic joints. Beside its function in the plasminogen activation process, the u-PA/u-PAR interaction also induces plasmin-independent events, such as chemotaxis and chemokinesis [14], the proliferation [15,16] and differentiation [17] of RA synoviocytes and the autocrine secretion of u-PA [17]. Recently, our group has shown that, in healthy synoviocytes, the u-PA/u-PAR interaction determines chemotaxis, chemoinvasion and proliferation in a dose-dependent fashion [18].

More recently, we have shown that RA synoviocytes over-express u-PAR and PAI-1, under-express u-PA, and are more prone than their normal counterpart to spontaneous and u-PA-challenged invasion and proliferation [19].

Raloxifene (RAL) is a selective estrogen receptor modulator (SERM) [20,21] with anti-estrogen activity in uterus and breast tissues and pro-estrogen activity in bone [22,23]. In post-menopausal women, RAL is widely used in the prevention and treatment of osteoporosis due to its anti-resorptive activity and established efficacy in reducing the risk of vertebral fracture [24].

Depending on the endocrine balance, synoviocyte activity can be reduced or enhanced, leading to amelioration or exacerbation of synovitis, respectively. These considerations provide a link between hormonal status and the mechanism of ECM destruction in RA and open new avenues for possible future therapeutic intervention. While the regulatory effect of estrogen is partly targeted to synoviocyte-associated MMPs and TIMPs [7], little is known about the effects of RAL on healthy and RA synoviocytes and their fibrinolytic pattern.

Therefore, our aim was to observe the effects of RAL on the fibrinolytic components (u-PA, u-PAR and PAI-1) of RA synoviocytes in order to modulate their levels and to reduce their fibrinolytic-dependent cellular proliferation and invasion.

## Materials and methods

### Patients

Sex and age matched patients with RA and healthy controls were used as a source of synoviocytes. Synovial tissue was obtained from four RA patients undergoing surgery for synoviectomy or joint replacement, and from four controls undergoing orthopaedic surgery for knee trauma. Informed consent was obtained from the patients enrolled in this study and ethics approval was given by the local ethics committee.

### Synovial cell cultures

Synovia was removed from knee joints, cut and subjected to a mild proteolytic treatment (0.05% trypsin, 0.5 mM EDTA in phosphate buffer saline, for 10 minutes at 37°C under gentle shaking). Trypsin was neutralized with FCS (Celbio, Milano, Italy) and cells were plated in culture dishes with RPMI 1640 (Cambrex Bio Science, Milano, Italy) supplemented with 10% FCS, 2 mM glutamine (Cambrex Bio Science) and penicillin-streptomycin (Cambrex Bio Science). Cell monolayers were used within the seventh passage in culture. The cells were considered type B fibroblast-like synoviocytes if negative by staining with anti-CD69, anti-CD14, anti-CD11b and anti-CD11c (Santa Cruz Biotechnology, Santa Cruz, CA, USA), positive by staining for the enzyme uridine-diphospho glucose dehydrogenase, and if they had a spindle-shaped, fibroblast-like morphologic appearance.

RAL (Lilly, Sesto Fiorentino (FI), Italy) was dissolved in methanol 100 mmol and further diluted with culture medium. Samples were analyzed both in basal conditions and after treatment with RAL (48 h). In preliminary experiments, we tested the effects of different doses of RAL in order to identify which dose would be more effective on synoviocytes without a lethal effect. Thus, the concentrations of 0.5 μM and 1 μM RAL were chosen for further experiments and used in this study. These concentrations were approved by the manufacturer (Lilly) and seem to be comparable with normal therapeutic doses, even if it is impossible to know which dose really reaches the synovia.

### A431 cell culture

A431 is a human epidermoid cancer cell line from a cervix squamous cell carcinoma. A431 cells were cultivated in DMEM with 4.5 g/L glucose (Cambrex Bio Science), supplemented with 10% FCS, 2 mM glutamine and penicillin-streptomycin. When cells were at confluence they were washed and incubated with DMEM 0.2% FCS for 48 h. Then the conditioned medium was centrifuged to remove particles and stocked at -80°C. It was used as a chemoattractant reagent in the chemoinvasion assay because of its known property to secrete chemoattractant soluble factors [25-27].

### Proliferation assay

Cell growth was quantified in subconfluent cell monolayers. Synoviocytes were seeded in 24 multi-well plates (15,000 cells/well) with 10% FCS in RPMI 1640. After 48 h incubation, cells were washed three times with serum-free medium and incubated in 0.2% FCS medium for an additional 48 h. Cells were then incubated for 48 h in 10% FCS medium (positive control); 0.2% FCS medium (negative control); 0.2% FCS with u-PA (Serono, Roma, Italy) 500 ng/ml with/without u-PA or u-PAR antagonists. The agonists were anti-human u-PA monoclonal antibody 5B4 (mAb 5B4) and anti-u-PAR monoclonal antibody 3936 (mAb 3936) (American Diagnostica, Montreal, Canada), which were used at 1.5 μg/ml. Both mAb 5B4 and mAb 3936 sterically impede the u-PA/u-PAR interaction and were developed against the u-PA-binding site of u-PAR. In preliminary experiments, we tested the effects of different doses of u-PA to identify what dose would be more efficacious in inducing synoviocyte proliferation. Thus, the concentration of 500 ng/ml u-PA was chosen.

Each experimental point was performed in triplicate. At the end of incubation cells were counted. Samples were analyzed both in basal conditions and after treatment with RAL 0.5 μM and 1 μM (48 h).

### Analysis of u-PA, u-PAR and PAI-1 levels

Samples were analyzed for u-PAR, u-PA and PAI-1 using commercially available ELISA kits (IMUBIND, American Diagnostica, Montreal, Canada) according to the manufacturer's instructions. Briefly, synoviocytes were seeded in six multi-well plates (25,000 cells/well) with 10% FCS in RPMI 1640. After 48 h of incubation, cells were washed three times with serum-free medium and incubated in 0.2% FCS medium for an additional 48 h. Cells were then treated with 0.5 μM or 1 μM RAL for 48 h. At the end of incubation, cells were detached, counted and lysed by a lysis buffer, as suggested by the manufacturer. The lysates were replaced in their original well and incubated for 1 h at 4°C to allow exhaustive extraction of undetached material. Cell extracts were centrifuged and stocked at -80°C until u-PAR analysis. Culture mediums were collected, centrifuged and stocked at -80°C until u-PA and PAI-1 determination. The results were correlated to the standard curve, within the range of linearity. Each sample was evaluated in triplicate and with two different dilutions. The u-PA assay reveals u-PA antigen, independent of its catalytic activity; the u-PAR and PAI-1 assays measure both occupied and unoccupied receptor, and both free and u-PA-coupled PAI-1, respectively.

The sensibility levels were: 10 pg of u-PA/ml of sample; 0.1 ng of u-PAR/ml of sample; 1 ng of PAI-1 /ml of sample.

### Analysis of u-PA enzymatic activity

u-PA enzymatic activity was evaluated by zymography. Culture medium samples were collected as described above and concentrated by centrifugation at 8,000 rpm for 30 minutes in centricon tubes (Amicon Division, Beverly, MA, USA) with 30 kDa molecular weight cut-off pores. The samples were subjected to SDS-PAGE (10%) under non-reducing conditions and migrated proteins were transferred onto 0.45 μm pore-size nitrocellulose filters (Bio-Rad Laboratories, Richmond, California, USA) in a 0.04 M phosphate buffer (pH 6.5) and run for 2 h under a current of 0.4 A. The nitrocellulose filter was removed and placed on an indicating layer containing casein and plasminogen. After overnight incubation at 37°C, u-PA digestion of plasminogen showed clear bands of lysis in the cloudy casein background, corresponding to the position of plasminogen activators in the polyacrylamide gel. u-PA 0.1 U/ml was used as a positive control. Zymograms were then scanned. Samples were analyzed both in basal conditions and after treatment with 0.5 μM or 1 μM RAL (48 h).

Because zymography was performed after protein separation by SDS-PAGE, which uncoupled u-PA from PAI-1, we used a direct fibrinolytic assay of cell membrane-associated plasminogen activators to study the final fibrinolytic balance of synoviocytes. Briefly, synoviocyte monolayers were treated with acidic wash (50 mM glycine-HCl buffer, pH 3.0, 0.1 M NaCl) to uncouple both u-PA and u-PA/PAI-1 complexes from cell surface u-PAR; Hepes buffer 0.5 M NaCl, pH 7.5, was added after 3 minutes. Aliquots of the acidic wash were dotted on fibrin plates prepared according to a method previously described [17] and fibrinolytic activity was evaluated after 16 h of incubation at 37°C, measuring the diameter of the lysis areas. Each sample was evaluated in triplicate.

### Radioiodination of u-PA and u-PA binding assay

u-PA was subjected to radioiodination with ^125^I-Na, using Iodogen (Pierce Eurochemie BV, Holland), following the instructions of the manufacturer. Iodinated protein was separated from non-incorporated radioactivity by gel filtration on Sephadex G-25 (Pharmacia, Milan, Italy). The specific activity obtained in the preparation used in this study was 17.8 μCi/μg. Saturation binding experiments were performed as previously described [28]. Binding experiments were performed either on untreated cell monolayers or following acidic wash (50 mM glycine-HCl buffer, pH 3.0, 0.1 M NaCl) to uncouple u-PA from cell surface u-PAR; Hepes buffer 0.5 M NaCl, pH 7.5, was added after 3 minutes.

### Migration assays

The Boyden chamber procedure was used to evaluate cell migration [14]. The method is based on the passage of cells across porous filters against a concentration gradient of the migration effector. A 48-well micro-chemotaxis chamber (Neuroprobe, Gaithersburg, MD, USA) was used. The two wells were separated by a polyvinyl-pyrrolidine-free polycarbonate filter with an 8 μm pore size (Neuroprobe). To evaluate chemoinvasion, the filter was coated with Matrigel (50 μg/filter) (Becton Dickinson Labware, Two Oak Park, Bedford, MA, USA). Test solutions were dissolved in serum-free medium and placed in the lower wells. 50 μl of cell suspension (12,500 cells) were added to each upper well. u-PA 100 ng/ml and/or RAL (0.5 μM or 1 μM) were added to the upper and/or lower well. In the experiment with neutralizing antibodies, the anti-u-PA mAb 5B4 (1.5 μg/ml) was placed in the lower wells, while the anti-u-PAR mAb 3936 (1.5 μg/ml) was incubated with the cell suspension. The chamber was incubated at 37°C for 5 h after which the filter was removed and fixed with methanol. Non-migrating cells on the upper surface of the filter were removed with a cotton swab. Cells were stained with Diff-Quick (Mertz-Dade AG, Dade International, Milan, Italy) and counted using a light microscope (40×) in 10 random fields for each well. Mobilization was measured by the number of cells moving across the Matrigel and the filter pores and spread on the lower surface of the filter. Each experimental point was performed in triplicate. Mean values of migrated cells for each experimental point were calculated.

### Statistics

A non-parametric Mann-Whitney test for independent samples was used to compare results from healthy and RA synoviocytes for the levels of u-PA, u-PAR and PAI-1. The results were expressed as mean ± standard deviation. The proliferation induced by u-PA and RAL was evaluated by two tailed t-test for independent samples and by analysis of variance (ANOVA with Bonferroni correction). Migration was measured as a percentage of the healthy basal response and was expressed as the mean ± standard error of the mean.

## Results

### u-PA-dependent proliferation of synoviocytes

**Figure 1:**
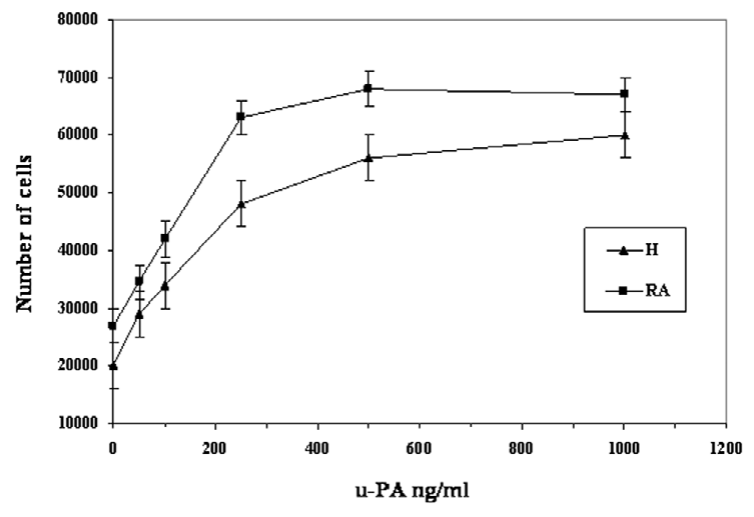
Urokinase-type plasminogen activator u-PA/u-PA receptor-dependent proliferation in healthy (H) and rheumatoid arthritis (RA) synoviocytes. Synoviocytes were seeded in 24 multi-well plates (15,000 cells/well) with 10% FCS in RPMI 1640. After 48 h incubation, cells were washed three times with serum-free medium and incubated in 0.2% FCS medium for an additional 48 h. At this time, cells were incubated for 48 h in 0.2% FCS containing increasing concentrations of u-PA (50 to 1,000 ng/ml). Proliferation of H and RA synoviocytes is reported as a function of u-PA concentration. Each point represents the mean ± standard deviation of three experiments performed in triplicate on four normal and four RA synovial cell lines.

u-PA induced a dose-dependent proliferation in both normal and RA synoviocytes, reaching a maximum at 500 ng/ml (p < 0.001 for any dose in both cell lines) (Fig. 1). Proportionally, u-PA increased cell proliferation in RA and healthy synoviocytes, without reaching a significant difference between the two cell lines at any u-PA dose.

These results show that u-PA has a pro-proliferative effect on both healthy and RA synoviocytes and identify 500 ng/ml u-PA as the minimal dose providing the maximal proliferative effect. This dose has been used in the following experiments on u-PA-dependent proliferation.

### Effects of Raloxifene on synoviocyte proliferation

**Figure 2:**
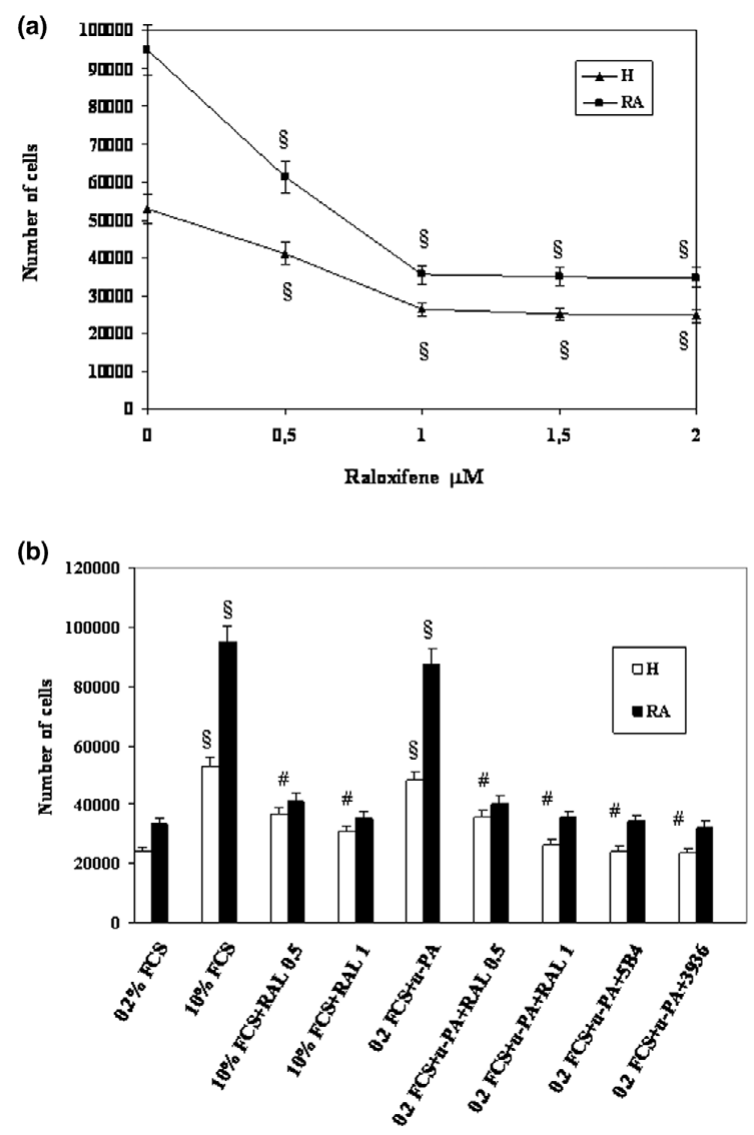
Effects of Raloxifene (RAL) on proliferation in healthy (H) and rheumatoid arthritis (RA) synoviocytes. **(a) **Proliferation of H and RA synoviocytes as a function of RAL concentration. Synoviocytes were seeded in 24 multi-well plates (15,000 cells/well) with 10% FCS in RPMI 1640. After 24 h incubation, cells were treated with increasing concentrations of RAL (0.5 to 2 μM). **(b) **Proliferation of H and RA synoviocytes under control conditions (0.2% FCS as a negative control, and 10% FCS as a positive control), in 0.2% FCS under the effect of 500 ng/ml urokinase-type plasminogen activator (u-PA) and in the presence of 0.5 and 1 μM RAL or the monoclonal antibodies (mAbs) 5B4 and 3936, which impair u-PA/u-PAR binding (see text for details). In both **(a) **and **(b)**, each point represents the mean ± standard deviation of three experiments performed in triplicate on four normal and four RA synovial cell lines. § = p < 0.01 versus basal (0.2% FCS); # = p < 0.001 versus 10% FCS or u-PA, for both H and RA.

RAL inhibited cell proliferation in a dose-dependent manner in both normal (p < 0.001) and RA synoviocytes (p < 0.001), reaching a maximal effect at 1 μM (Fig. 2a). The RAL induced decrease in cell proliferation did not significantly differ between RA and healthy synoviocytes for any RAL dose. These results indicate that RAL exerts an anti-proliferative effect on both healthy and RA synoviocytes that is effective at doses as small as 0.5 μM.

### Effects of Raloxifene on u-PA-dependent proliferation

In both normal and RA synovial cells, RAL significantly reduced u-PA- and 10% FCS-dependent proliferation (p < 0.001) (Fig. 2b). RA synoviocytes are as prone as normal synoviocytes to spontaneous (0.2% FCS) proliferation and to proliferation challenged both with 10% FCS and 500 ng/ml u-PA (Fig. 2b). In particular, serum-dependent proliferation (10% FCS) did not significantly differ from u-PA-dependent proliferation in both healthy and RA cell lines.

The proliferative effect elicited by 500 ng/ml of u-PA in 0.2% FCS was significantly reduced by the mAb antagonists of u-PA (mAb 5B4) and u-PAR (mAb 3936) in healthy (p < 0.001) and RA synoviocytes (p < 0.001) (Fig. 2b). This indicates that u-PA/u-PAR interaction is required for the pro-proliferative effect of u-PA on normal and RA synoviocytes.

RAL blocked both FCS-dependent and u-PA-dependent proliferation to a similar extent. RAL reduction of u-PA-dependent proliferation was similar to the inhibition caused by the antagonist antibodies (p < 0.001 in normal and RA synoviocytes) (Fig. 2b) and was not increased by co-treatment with them, indicating a common target for RAL and anti u-PA/u-PAR antibodies (data not shown). This suggests that inhibition of the expression of critical members of the cell-associated fibrinolytic system is a likely target of RAL treatment.

### u-PA, u-PAR and PAI-1 levels in normal and RA synoviocytes

**Figure 3:**
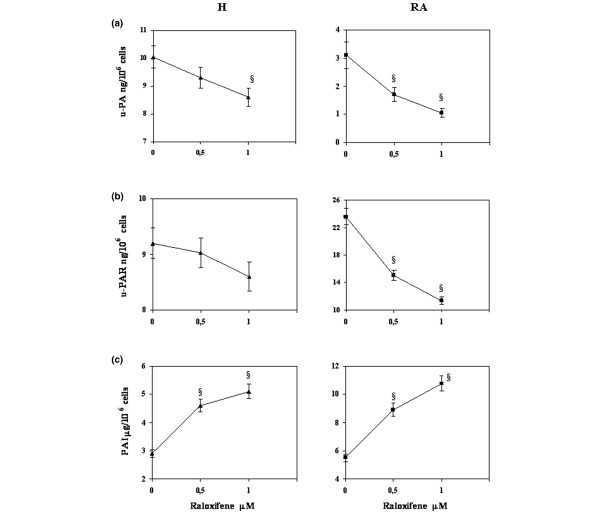
Fibrinolytic pattern of healthy (H) and rheumatoid arthritis (RA) synoviocytes treated with Raloxifene. Samples were analyzed both in basal conditions and after treatment with 0.5 and 1 μM Raloxifene (48 h). **(a) **Urokinase-type plasminogen activator (u-PA) antigen was quantified on aliquots of the culture medium by ELISA assay and reported as ng/10^6 ^synovial cells. **(b) **u-PA receptor (u-PAR) was quantified by ELISA assay on aliquots of cell lysates and reported as ng/10^6 ^synovial cells. **(c) **Plasminogen activator inhibitor (PAI)-1 was also measured in aliquots of the culture medium, and reported as μg/10^6 ^synovial cells. H refers to four different synovial cell cultures from healthy individuals, RA refers to synovial cell cultures from four different rheumatoid arthritis patients. In **(a-c)**, each point represents the mean ± standard deviation of three experiments performed in triplicate on each synovial cell line. § = p < 0.01 versus basal (0.2% FCS).

As our ELISA assay for u-PA measures u-PA antigen independent of its interaction with PAI-1, the data shown in Fig. 3a indicate that u-PA released into the culture medium by RA synoviocytes was significantly lower than u-PA produced by healthy synoviocytes (3.1 ± 0.4 versus 10.05 ± 0.04 ng/10^6 ^cells, respectivle; p < 0.05).

**Figure 4:**
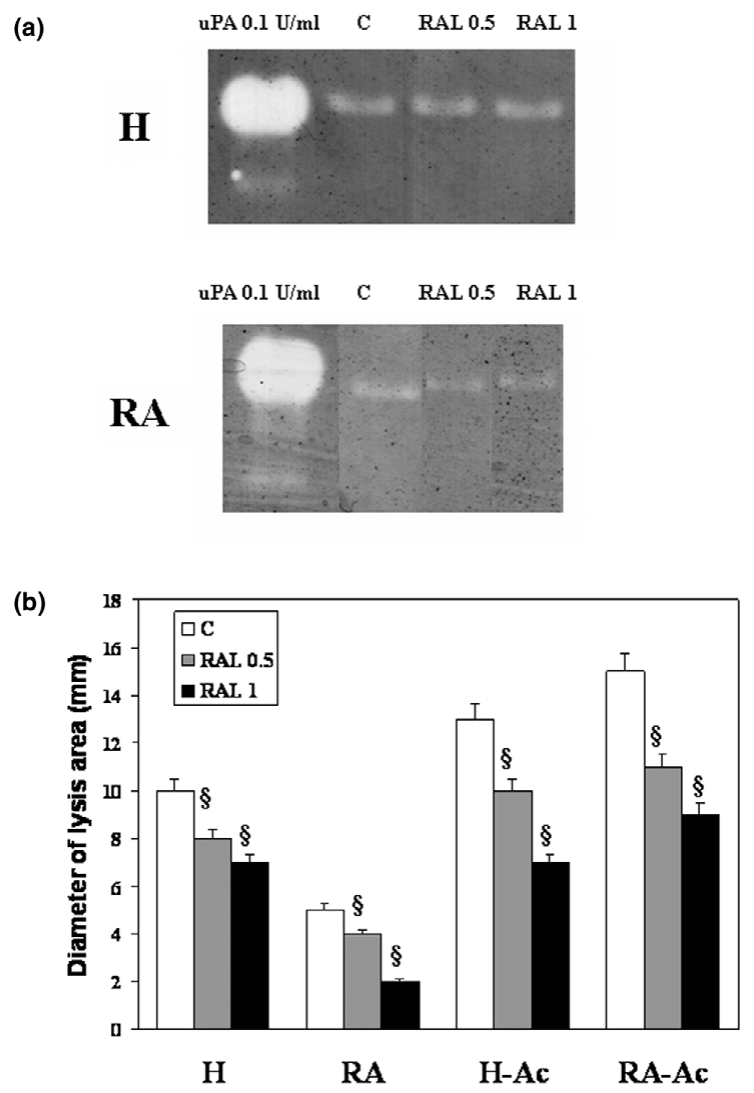
Urokinase-type plasminogen activator (u-PA) activity in healthy (H) and rheumatoid arthritis (RA) synoviocytes treated with Raloxifene (RAL). Samples were analyzed both in basal conditions and after treatment with 0.5 and 1 μM RAL (48 h). **(a) **Zymographic assay of aliquots of the culture medium. u-PA digestion of plasminogen shows clear bands of lysis in the cloudy casein background of the indicating layer. Shown here are the effects on one H and one RA line; the other lines furnished similar results. **(b) **Direct fibrinolytic assay of cell membrane-associated plasminogen activators. Left side: lysis areas of culture medium under the indicated experimental conditions. Right side: lysis areas of aliquots of the acidic wash (Ac), indicating the activity eluted from the cell surface. The diameter (mm) of the lysis areas were measured as an index of fibrin digestion. H refers to four different synovial cell cultures from healthy individuals; RA refers to synovial cell cultures from four different RA patients Each point represents the mean ± standard deviation of three experiments performed in triplicate on each synovial cell line. § = p < 0.01 versus control (C).

A zymographic assay of u-PA performed on aliquots of the culture medium confirmed the data obtained using antibodies. Culture mediums of RA synoviocytes display a lower u-PA activity than medium obtained from healthy synoviocytes (Fig. 4a).

The direct fibrinolytic assay, performed on fibrin plates as described, indicated the following: the culture medium of healthy synoviocytes showed a higher fibrinolytic activity than RA synoviocytes, thus confirming the data obtained with zymography, again indicating lower constitutive u-PA production in RA synoviocytes (Fig. 4b, left side); and the fibrinolytic activity exhibited by aliquots of the acidic wash was slightly higher in RA than in healthy synoviocytes (Fig. 4b, right side). This means that, regardless of the lower amount of secreted u-PA, a larger availability of u-PAR on the surface of RA synoviocytes enabled RA synovial cells to bind a higher amount of the ligand. These results have also been confirmed by radioligand binding experiments. Healthy synoviocytes exhibited 95 ± 15 × 10^3 ^u-PAR/cell, with a Kd ranging from 1.75 to 1.85 nM, and the receptor number was 140 ± 25 × 10^3^/cell following acidic treatment prior to performing binding experiments. RA synoviocytes had 180 ± 30 × 10^3 ^uPAR/cell before and 230 ± 48 × 10^3 ^u-PAR/cell after acidic wash, while the Kd did not change. Higher levels of u-PAR are found in cell lysates of RA than of healthy synoviocytes (23.6 ± 0.10 versus 9.2 ± 0.05 ng/10^6 ^cells, respectively; p < 0.05), also determined using ELISA (Fig. 3b).

Moreover, RA synovial cells release into culture medium higher amounts of PAI-1 than healthy synoviocytes (5.5 ± 0.1 versus 2.9 ± 0.1 μg/10^6 ^cells, respectively; p < 0.05; Fig. 3c).

### Effects of Raloxifene on u-PA, u-PAR and PAI-1 levels in normal and rheumatoid arthritis synoviocytes

RAL reduces u-PA levels in healthy and RA synoviocytes (p < 0.0001; Fig. 3a). It does this dose dependently: 1 μM RAL is more efficient than 0.5 μM in healthy (p < 0.01) and in RA synoviocytes (p < 0.05). RAL-dependent reduction of u-PA levels at 0.5 μM and 1 μM is significantly higher (p < 0.05) in healthy than in RA synoviocytes.

A zymographic assay performed on culture medium treated with 0.5 μM and 1 μM RAL confirmed the data obtained with the u-PA assay. In fact, RAL reduces the u-PA enzymatic activity in a dose dependent manner, in both healthy and RA synoviocytes (Fig. 4a).

The direct fibrinolytic assay on fibrin plates, performed with aliquots of culture medium, indicated that RAL-dependent reduction of cell-associated fibrinolytic activity is more pronounced in RA than in normal synoviocytes (Fig. 4b, left side). Fig. 4b (right side) shows a RAL-dependent reduction of u-PA in the acidic wash of both normal and RA synoviocytes.

RAL significantly reduces u-PAR levels in cell lysates from RA synoviocytes (p < 0.0001; Fig. 3b); 1 μM RAL is more efficient than 0.5 μM at reducing u-PAR levels in healthy and RA synoviocytes (p < 0.01). The effect of 0.5 μM and 1 μM RAL is significantly higher in RA than in healthy synoviocytes (p < 0.05). Radioligand binding experiments showed that, upon RAL treatment, the receptor number before and after acidic treatment was similar in healthy synoviocytes, whereas a dramatic decrease of both unoccupied u-PAR (85 ± 18 × 10^3 ^receptor/cell, before acidic wash) and total u-PAR (150 ± 25 × 10^3 ^receptor/cell after acidic wash) was observed in RA synoviocytes.

RAL significantly increases PAI-1 levels in healthy and RA synoviocytes (p < 0.0001; Fig. 3c). This is true for both 0.5 μM and 1 μM RAL (healthy synoviocytes, p < 0.01 and p < 0.001, respectively; RA synoviocytes, p < 0.001 and p < 0.01, respectively). RAL 1 μM is more efficient than RAL 0.5 μM at increasing PAI-1 levels in both healthy and RA synoviocytes (p < 0.001). RAL 0.5 μM and 1 μM have a significantly higher effect on increasing PAI-1 levels in RA than in healthy synoviocytes (p < 0.05).

### u-PA-dependent synoviocyte chemoinvasion

**Figure 5:**
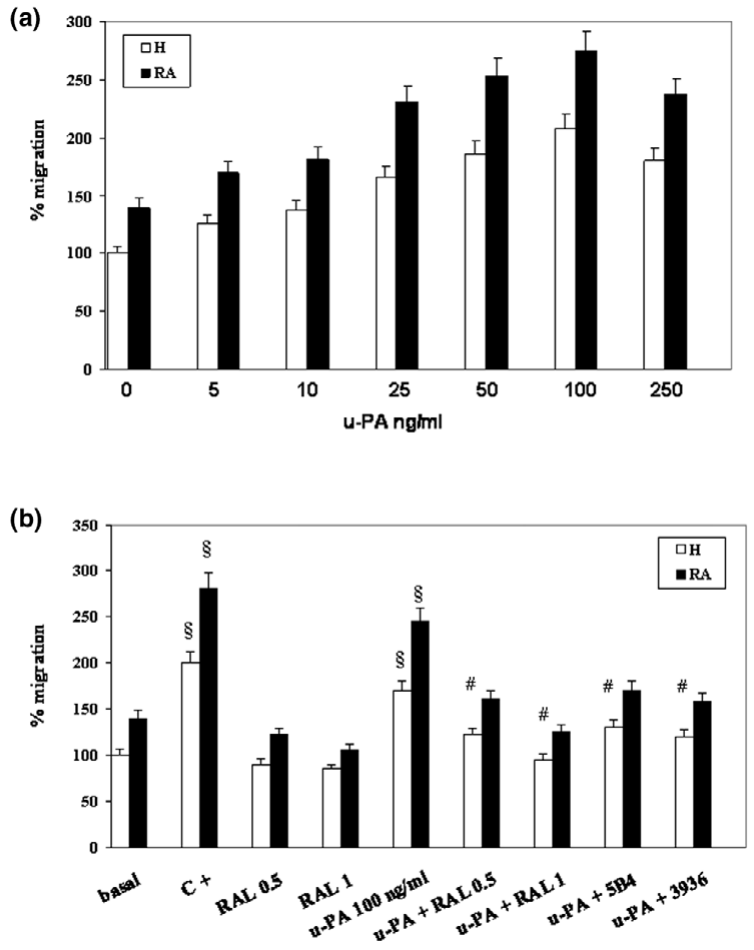
Urokinase-type plasminogen activator (u-PA)/u-PA receptor (u-PAR)-dependent chemoinvasion in healthy (H) and rheumatoid arthritis (RA) synoviocytes. **(a) **Cell invasion of Matrigel-coated filters by H and RA synoviocytes as a function of u-PA concentration. Migration was stimulated by increasing concentrations of u-PA (5 to 250 ng/ml) in the lower well of the migration chamber. **(b) **Percent increase of Matrigel invasion in H and RA synoviocytes treated with Raloxifene (RAL) or neutralizing antibodies. Basal, invasion of H and RA synovial cells in the presence of 0.2% FCS in the lower well; C+, invasion stimulated by conditioned medium of A431 cell line, used as a sure chemotactic agent; RAL 0.5 and RAL 1, invasion challenged with 0.5 μM and 1 μM RAL in the lower well; u-PA, invasion challenged with 100 ng/ml of u-PA in the lower well; u-PA+RAL 0.5 and u-PA+RAL 1, invasion challenged with 100 ng/ml u-PA in the presence of 0.5 and 1 μM RAL in the lower well; u-PA+5B4 and u-PA+3936, invasion challenged with 100 ng/ml u-PA in the presence of 1.5 μg/ml of monoclonal antibodies 5B4 and 3936 in the lower and upper well, respectively. In **(a) **and **(b)**, each point represents the mean ± standard deviation of three experiments performed in triplicate on four normal and four RA synovial cell lines. § = p < 0.01 versus basal (0.2% FCS); # = p < 0.001 versus u-PA, for both H and RA.

u-PA-dependent chemoinvasion was dose-dependent (Fig. 5a), with a maximal effect at 100 ng/ml for both healthy and RA synoviocytes. Basal migration was more pronounced (more than 40%) in RA than in healthy synoviocytes. This difference is maintained at each concentration of u-PA in the lower well of the migration chamber. An increase in invasion observed after a 5 h incubation with 100 ng/ml u-PA was counteracted by the incubation of invasive cells with mAb antagonists of u-PA (mAb 5B4) and u-PAR (mAb 3936) (Fig. 5b). This indicates that u-PA/u-PAR interaction is required for the pro-invasive effect of u-PA on normal and RA synoviocytes.

### Effects of Raloxifene on u-PA-dependent synoviocyte chemoinvasion

RAL inhibits cell chemoinvasion in a dose-dependent manner in both normal and RA synoviocytes, reaching a maximum at 1 μM (Fig. 5B); this decrease did not significantly differ between healthy and RA cells at any RAL dose.

RAL significantly reduced u-PA-induced chemoinvasion in healthy and RA synoviocytes; 1 μM RAL reduced chemoinvasion to basal values. When cells were incubated together with RAL in the upper chamber, it caused similar effects on chemoinvasion. In fact, RAL was able to inhibit migration induced by u-PA in a dose-dependent way with a maximal action at a dose of 1 μM and this decrease in mobility was proportionally similar in healthy and RA synoviocytes (data not shown).

Upon combining the results shown in Figs 3, 4 and 5, one is tempted to think that RAL works mainly through the reduction in the levels of u-PAR and, therefore, by blocking u-PA-dependent chemoinvasion. In fact, the RAL reduction of u-PA-dependent migration was similar to the inhibition caused by the antagonist mAbs (Fig. 5b) and is not increased by co-treatment with them, indicating that RAL and anti-u-PA/u-PAR antibodies have a common target (data not shown).

## Discussion

This is the first study on the effects of RAL on RA synoviocytes and on their fibrinolytic pattern and function. The presence of functional estrogen receptors (ER-alpha) in synoviocytes has been identified [7,29]. Our data show that RAL acts as an inhibitor of the fibrinolytic system, exhibiting similar effects on both healthy and RA synoviocytes, with the activity on RA synoviocytes being more pronounced. Indeed, the drug inhibits cell proliferation and chemoinvasion in a dose-dependent manner, reduces u-PA and u-PAR levels, increases PAI-1 levels and blocks u-PA by acting on the specific interaction between u-PA and u-PAR.

Despite increasing evidence on the significance of sex hormones in RA, their etiopathological role and potential long term effect on RA progression still remain unclear. Also, the link between sex hormones and the fibrinolytic system is still to be elucidated in RA synovial cells.

In human breast cancer cells, estradiol inhibited the expression and secretion of u-PA, t-PA and PAI-1 proteins. Zymography confirmed the inhibitory effect of estradiol on u-PA activity [30]. The regulation of expression of genes encoding u-PA and PAI-1 by estradiol and different SERMs has been described in human breast cancer cells [31]. Two different SERMS (4-hydroxytamoxifen and RAL) had a concentration-dependent agonistic (estradiol-like) effect on the regulation of these genes. In contrast, a pure anti-estrogen alone had no effect but could block the action of estradiol and SERMs. This demonstrates that RAL has an estrogen-like effect on human breast cancer cells with respect to the regulation of u-PA and PAI-1 gene expression [31].

These findings prompted us to investigate the effects of RAL on the fibrinolytic system of RA synovial cells. In fibroblast-like synoviocytes, we found a link between the functionality of another proteolityc system (the u-PA/u-PAR-PAI-1 system) and a SERM such as RAL. In RA, the formation and invasiveness of synovial pannus, supported by angiogenesis, is linked to serine proteinases, mainly u-PA [11], produced in high quantity by synoviocytes [12,18,19].

The u-PA/u-PAR-PAI-1 system is an organizer of cell-ECM contacts and covers the full range of activities required to promote invasion and angiogenesis and to disrupt cell attachment sites. We have recently shown that RA synoviocytes display a fibrinolytic machinery (u-PA, u-PAR and PAI-1) addressed toward an invasive pattern [19]. RAL inhibits the proliferation of RA synoviocytes dose dependently and blocks the proliferation induced by u-PA, mainly by reducing u-PAR levels without affecting the Kd of the u-PA/u-PAR interaction (as shown in this study by ELISA and radioligand binding experiments) and, therefore, by reducing the specific interaction between u-PA and u-PAR following reduction of u-PAR.

Cellular migration, linked to cellular adhesiveness, is an important process for the invasion of articular cavity and extra-articular tissues, typical of RA. Our data show that RAL inhibits migration of RA synoviocytes, thus potentially contributing to the modulation of the growth of synovial pannus. u-PAR regulates pericellular proteolysis and cell surface adhesion receptors, fundamental events in the first steps of invasion and angiogenesis. RAL further reduces u-PA levels derived from RA synoviocytes, thus potentially reducing the invasive and angiogenetic potential of these cells.

The increased expression of PAI-1 in RA synoviocytes is in agreement with previous data showing higher production of PAI-1 in cultured RA synoviocytes [12,19]. In RA, elevated PAI-1 levels could act as an ECM-stabilizing molecule by blocking extracellular proteolysis, thus providing cells with a substrate favoring cell movement. At the same time, PAI-1 promotes cell detachment [32]. RAL increases PAI-1, thereby blocking the u-PA-dependent ECM degradation and reducing cell movement. The whole *in vitro *scenario is that RAL reduces u-PAR and u-PA levels and increases PAI-1 levels. This action may significantly contribute to reducing the pro-invasive and pro-angiogenic pattern of RA synoviocytes, although the clinical relevance of these findings require further study.

The over-expression of u-PAR in RA may depend on the need to activate the fibrinolytic pathway in order to degrade and invade ECM, as well as to promote interaction between u-PAR and vitronectin, which provides the adhesive grip necessary for cell locomotion, events required in all the invasive pathologies [33]. RAL seems to exert an upstream blocking of the membrane-bound fibrinolytic system, which also stops the proteolytic activities of MMPs.

## Conclusion

RAL modulates *in vitro *the components and functionality of the membrane-bound fibrinolytic system, exhibiting a more intense activity in RA synoviocytes compared to their normal counterpart. RAL inhibits u-PA-induced proliferation and u-PA-induced cell migration dose dependently. The effect of RAL in the reduction of the formation of synovial pannus and the radiological progression of RA warrants further study.

## Abbreviations

DMEM = Dulbecco's modified Eagle medium; ECM = extracellular matrix; ELISA = enzyme-linked immunosorbent assay; FCS = fetal calf serum; mAb = monoclonal antibody; MMP = matrix metalloproteinase; PAI = plasminogen activator inhibitor; RA = rheumatoid arthritis; RAL = Raloxifene; SERM = selective receptor estrogen modulator; TIMP = tissue inhibitor of matrix metalloproteinase; u-PA = urokinase-type plasminogen activator; u-PAR = urokinase-type plasminogen activator receptor.

## Competing interests

The authors declare that they have no competing interests.

## Authors' contributions

SG isolated synoviocytes, carried out the chemoinvasion assay and participated in drafting the manuscript. ADR participated in drafting the manuscript and performed the statistical analysis. MC carried out the ELISA assay, proliferation experiments and participated in drafting the manuscript. RL performed the fibrinolytic assay. FP, AR, AG, RG and NI participated in the design of the study. GF and MDR coordinated the study. MMC conceived the study.
